# London Letter

**Published:** 1888-01

**Authors:** Wm. B. Meany

**Affiliations:** London, England


### FOREIGN CORRESPONDENCE.

*To the Editor of the Chicago Medical Journal and Examiner:*

THE NEW PRODUCT.

The fact that a substance two hundred and twenty times sweeter than sugar is abstracted by an abstruse series of chemical processes from such an uncleanly looking product as coal-tar, seemed, when first bruited, a flight of imagination, and not worth being considered as a commercial reality. But such it has become. *Saccharine* is not now only a pretty laboratory experiment, as it was first thought to be. It was even stated that for commercial and domestic purposes it was impossible. To chemists now *saccharine* as a commercial product is a fact, pure and simple.

Saccharine has sweetening properties, but it is not sugar. It will sweeten but not fatten; it has no action whatever, as far as yet ascertained, upon the human system. Professor Stotzer, of Bonn, has conducted a variety of experiments upon animals, and affirms that the effect is *nil* on the canine system, although as much as would equal a pound of sugar has been administered daily. However, on the other hand, it is a pure chemical compound, and, it is said, may in time produce certain changes, especially in the liver.

It is made from the toluene of coal-tar, which is first treated with sulphuric acid in closed vessels rotating on horizontal axles. After the toluene has disappeared, the contents are run off in tanks, cold water and chalk being added. The product then contains calcium, ortho- and para-toluene sulphonates. This again is treated with carbonate of sodium and allowed to cool after the sodium salts have evaporated. The dried sulphonates then undergo a mixing with phosphorus tri-chloride, placed in lead-lined vessels, and chlorine is passed over the mixture. The oxy-chloride is driven off and phosphate of lime precipitated. When the sulpho compounds are cooled, crystals of para- and liquid ortho-toluene sulpho-chloride appear, the latter of which, only, is capable of yielding saccharine.

The liquid is then treated with solid ammonium carbonate, and subjecting the mass to the action of steam, which sets the carbonic acid free. Permanganate of potash is added to oxidize it, and precipitation takes place by adding hydrochloric or sulphurous acid—the true *benzoic sulphinide* is formed.

It is stated that one grain will sweeten a cup of tea or coffee, and the most nauseous compound is made palatable by its use.

A NEW SYSTEM OF TREATMENT.

It is again asserted from Vienna that the Crown Prince of Germany has for some weeks been under a new system of treatment. That new treatment is based upon investigations of Dr. Ernest Freund, a Vienna disciple of Professor Ludwig. Dr. Freund, it is stated, began to devote himself specially to the study of cancer in 1885. He has published a treatise based upon seventy different cases of carcinoma, in all of which he found the chief characteristic to be an abundance of sugar in the blood, and he even fixed upon a certain relative quantity of sugar in the blood, as the condition for the formation of carcinomatous cellules. From such a theory it follows that patients suffering from cancer should abstain from all sugar-forming foods, and, in addition, Dr. Freund uses a medicament for the oxidation of superfluous sugar in the body. It is stated that this treatment is now being tried upon the Crown Prince of Germany with satisfactory effect, the growth in the larynx showing a constant tendency to diminish.

### CANCER AND VACCINATION.

The following table, which has recently been compiled, will show the strides made by cancer in England and Wales concurrently with the extension of vaccination, which was first made compulsory in 1853.

*Deaths from Cancer in England and Wales.*

| Decades. | Deaths. | Mean Annual Mortality per Million Living. |
|---|---|---|
| 1851–60 | 60,196 | 317 |
| 1861–70 | 82,371 | 387 |
| 1871–80 | 114,405 | 473 |
| 1881–84 (4 years) | 57,411 | 535 |

### M. PASTEUR'S NEW SUGGESTION.

M. Pasteur, I see, has come forward with a suggestion for the abolition of the Australian rabbit plague. He says that the best way to get rid of the rabbits is to create among them some disease which will spread among the animals; that it is of no avail to confine operations to methods by which one rabbit has to be killed at a time, but the rabbits themselves must help in their own extermination, so to speak. M. Pasteur's idea is to sprinkle water previously mixed with "microbes of chicken cholera" near the burrows or the run of these rodents. He thinks that the rabbits in this way would be certain to get the disease, and all others would be infected by it. Chicken cholera, he states, is fatal to rabbits. The poultry must be kept clear of these infected spots.

### SYSTEM OF ISOLATION.

London may at last feel reasonably confident that the epidemic of scarlet fever which existed during the summer months has exhausted its violence. London has utilized in the outburst of scarlet fever the buildings provided by the *Metropolitan Asylum Board*. This board has let it be understood that a refuge was ready for any who could not, with safety to themselves or to others, be nursed at home. It has been demonstrated here that isolation is the specific for the abridgement and the extinction of an epidemic of scarlet fever. It has been found that isolation in a room, or a couple of rooms, crowded with inmates, is unattainable.

By the establishing of a number of hospitals for the treatment of scarlet fever, the public began to see the advantages, both to the sick and the well, of the excellent nursing and judicious treatment.

Many patients of the well-to-do classes are known to have entered these hospitals. Had the old condition of things existed London would now, no doubt, still be a fever-stricken city, as relatives, friends, attendants and fellow lodgers would have been perpetually distributing the contagion.

### WOOL-SORTER'S DISEASE.

The following brief history of two recent cases of anthrax, or "wool-sorter's disease," may prove interesting to your readers.

CASE I. The patient, a male, 40 years old, was admitted to Guy's Hospital, complaining of feeling sick and of having a small pimple on the back of his neck, which was troublesome. He stated that he was employed at London Wharf to assort hides, etc. The small pimple had become greatly enlarged in the space of twenty-four hours, involving the glands of his neck, which were much swollen. The house-surgeon stated that on careful examination of the patient it was discovered that he was suffering from a malignant pustule on the neck, or, as it was known in the leather trade, "wool-sorter's disease." The patient sank rapidly, and died on the fourth day after his admission to the hospital.

Microscopical examination of the blood being made, it was established that the patient died from anthrax.

CASE II. The wife of a wool-sorter called to consult her physician for a swollen condition of the lids of the right eye. There was noticed on the right cheek, about an inch and a half below the eye, an inflamed spot about the size of a pea. This spot or pimple was very annoying, on account of a constant itching. She had no pain and did not feel ill. Her temperature was normal, but the pulse was rapid. She was visited by her physician at her house, accompanied by a consultant, on the evening after her morning visit.

The swelling had extended downward to the ear and jaw, upward and forward across the face, slightly towards the left eye. She complained of dizziness, and said she felt the blood rushing to her head, and was somewhat delirious. Her pulse, temperature and respiration were nearly normal. She was seen again the next morning, when she stated that she had passed a good night, had slept well, and felt no pain. The swelling had extended over the face generally. On the second day from her visit to the physician, her pulse was found to be varying—when lying in bed it was 96, when erect, 116, and after trying the vomit it went up to 144. The swelling had by this time extended over the head and around the neck. She complained of a choking sensation, and said she felt very tired. On the third day marked improvement appeared to have taken place in her general condition; there was some diminution of the swelling. The physician was summoned on the morning of the fifth day, and found her in a dying condition. This was the last time he saw her alive. The foregoing history of the case was kindly furnished me by her medical attendant, to whom I am also indebted for an invitation to witness the post mortem, which was held thirty-four hours after death. Externally there was a deep purple discoloration of the face, neck, and upper part of the chest, extending down the arms to the elbow joints. The fingers, limbs, and feet were of a dark color. The head, face and front part of the chest were much swollen. The right cheek and eyelids, when stripped of the skin, presented a grayish appearance. On opening the chest, the lungs were found adherent to the chest walls. There was nearly a pint of clear fluid in the pleural cavites. The lungs were of a darker color than natural. Except the spleen, the internal organs appeared healthy. The spleen was enlarged and "pulpy." The blood was very fluid and of a purplish color. On examination of specimens of the blood from the body there were found specific organisms characteristic of anthrax.

The deceased had always appeared to those who were acquainted with her to have been a strong and healthy woman. From the statement made by the patient, the poison was thought to have been inoculated into the spot on the cheek not more than ten days before her death.

Is it not possible that what has been effected in the case of the lower animals, as by Pasteur's inoculation, may ultimately be applied to the saving of human life? Is there any reason why inoculation with attenuated solutions may not be advantageously practiced upon those persons whose duties require their frequent handling of hides, wool, ect.?

WM. B. MEANY, M. D.

LONDON, ENGLAND.

